# Oral immunotherapy improves the quality of life of adults with food allergy

**DOI:** 10.1186/s13223-024-00915-6

**Published:** 2024-10-14

**Authors:** Na’ama Epstein-Rigbi, Michael B. Levy, Liat Nachshon, Yael Koren, Michael R. Goldberg, Arnon Elizur

**Affiliations:** 1 The Institute of Allergy, Immunology and Pediatric Pulmonology, Shamir Medical Center (Former Assaf Harofeh), 71300 Be’er Ya’akov, Israel; 2 Department of Pediatrics, School of Medicine, Tel Aviv University, Tel Aviv, Israel; 3 Department of Medicine, School of Medicine, Tel Aviv University, Tel Aviv, Israel

**Keywords:** Adults, Food allergy, Oral immunotherapy, Quality of life

## Abstract

**Background:**

Oral immunotherapy (OIT) has become the standard of care for children with food allergy (FA) and has substantially improved their quality of life. The effect of OIT on the quality of life in adults, however, has been studied to a much lesser degree.

**Methods:**

Patients with food allergy aged ≥ 18 years who underwent OIT at Shamir Medical Center completed the Food Allergy Quality of Life Questionnaire-Adult Form (FAQLQ-AF) before and at the end of treatment. Adults with FA not undergoing OIT who completed the FAQLQ-AF at 2 time points, served as controls.

**Results:**

A total of 44 adults, median age 23.4 years, who underwent OIT for milk (n = 19), egg (n = 2), peanut (n = 9), sesame (n = 6), and tree nuts (n = 8), and 11 controls were studied. The median OIT starting dose was 23.8 mg protein. 33 patients (75%) reached full desensitization within a median of 10.3 months. The FAQLQ-AF baseline scores were comparable between the study and control groups for all items except for Food Allergy related Health (FAH) item in which the study group had a significantly better score (p = 0.02). At the second time point, the study group had significantly better scores in all items (Allergen Avoidance and Dietary Restrictions (AADR), p = 0.02; and Emotional Impact (EI), Risk of Allergen Exposure (RAE), FAH and the Total Score, p < 0.01). The change in scores for the study group was significantly better, statistically and clinically, in AADR, p = 0.04; EI, p < 0.01; RAE, p = 0.01, and in the total score, p = 0.01.

**Conclusions:**

OIT significantly improves quality of life of adults with FA. This finding adds important support for providing OIT in this population.

**Supplementary Information:**

The online version contains supplementary material available at 10.1186/s13223-024-00915-6.

## Background

Food allergy (FA) has become a worldwide problem leading to significant social and economic burdens [1–3]. Oral immunotherapy (OIT) is successful in desensitizing most children with FA [4–6], improving patient and caretaker quality of life [7, 8], and gradually gaining worldwide recognition as a treatment modality [9, 10]. Recently, data published showed high desensitization rates for adults treated with egg, sesame, peanut and tree-nut OIT, but less so for milk [11]. However, outcomes of OIT should not just be limited to the achievement of desensitization, but should additionally focus on the changes to the patients’ anxiety level, social interactions, food avoidance and economic burdens. These considerations taken together represent the quality of life (QoL) of patients.

While the change in QoL of children following OIT focuses on a significant improvement for both patients and their caretakers [12–14], adults, face different challenges. The OIT treatment may lead to a more stressful and demanding daily routine and they may not necessarily have an immediate family member or other adult for support in case of an allergic reaction. Finally, they may be burdened with other personal or professional challenges, such as balancing between independent health management decisions to work related obligations, and traveling and caring for others [15]. These multitude of factors may impact on the adult’s QoL perception.

Since April 2010, the Institute of Allergy, Immunology and Pediatric Pulmonology at the Shamir Medical Center, has provided OIT to patients over the age of ≥ 4 years, including adults, with allergies to milk, egg, sesame, peanut, and tree nuts [16–21]. In this study, we examined the impact of OIT on the adult population, using the Food Allergy Quality of Life Questionnaire-Adult Form (FAQLQ-AF), developed to evaluate Health related QoL (HRQOL) in adults with food allergy [22].

## Methods

### Study population

Patients aged 18 years and above, with IgE-mediated food allergy, who underwent an open label OIT treatment program to milk, peanut, egg, sesame or tree nuts (walnut, cashew, hazelnut) at the Yitzhak Shamir (Assaf Harofeh) Medical Center between April 2014 and December 2022, were evaluated prospectively using FAQLQ-AF questionnaires. Data included only patients who reached a final disposition either of full desensitization, partial desensitization or treatment failure. Patients who were in their up-doing stage, were excluded. Adult patients with food allergy not undergoing OIT who filled the FAQLQ-AF at two separate time points were used as controls. Informed consent for treatment was obtained from all participants. Approval for the documentation and publication of patient data was obtained from the institutional ethics review board committee.

*Oral immunotherapy*. The OIT program at the Shamir Medical Center is individualized, performed in an ambulatory care setting. At the start of the program IgE-mediated food allergy was diagnosed by evidence of IgE sensitization, either by skin prick test (SPT) and/or specific serum IgE, together with a positive oral food challenge (OFC), or a clinical history of objective reactions following accidental ingestion in the past year. The diagnosis of previous anaphylaxis was defined as any reaction involving 2 or more organ systems and was based on the history obtained from the patients or their caretakers. A minimal tolerated dose of 5 mg protein for milk and of 1 mg in the case of all other foods was required to initiate OIT. Asthma was diagnosed using clinical history combined with evidence of reversible airflow obstruction on spirometry (Minispir, Mir, Rafamedical, Yavne, Israel). Patients with uncontrolled asthma, active eosinophilic gastrointestinal disease or autoimmune disease were excluded. Patients with stable asthma and those previously hospitalized for severe anaphylactic reactions were included. The program begins with a 3-to-4-day dose-escalation phase during which the dose eliciting a reaction in each patient is established, and the single highest tolerated dose (SHTD) is determined. During the first up-dosing round, all patients provide a thorough medical history, and undergo skin prick tests to the treated food and to House Dust Mite (HDM). Spirometry is performed in all patients prior to treatment, and in all patients with asthma during each up-dosing clinic visit. After each round, the achieved dose is consumed once daily at home, for 24 days, until the next visit. During the second and third up-dosing rounds the dose is escalated up to a maximum of fourfold. Treatment subsequently consists of alternate cycles of up-dosing and home-treatment phases until the food-specific target dose for partial or full desensitization is reached. Partial desensitization target doses (milk 180 mg protein; peanut 300 mg; tree nuts 300 mg; egg, 1500 mg, and sesame 240 mg protein) reflect a significantly increased protection in the case of accidental exposure, while full desensitization target doses (milk 7200 mg protein; egg 6000 mg; peanut 3000 mg; tree-nuts and sesame 4000 mg protein) enable free consumption [11]. Patients report on doses taken at home and any adverse reactions via a web-based reporting system [23].

### Food allergy quality of life questionnaires-adult form (FAQLQ-AF)

The FAQLQ-AF is a disease specific health related quality of life questionnaire, designed to evaluate the self- perceived QoL in adults with food allergy [22]. It consists of 29 items answered using a seven-point response scale (1 = no impairment;7 = most impairment), which are divided into four domains, combined together to generate a QoL total score: allergen avoidance and dietary restrictions (AADR), emotional impact (EI), risk of accidental exposure (RAE) and food allergy related health (FAH). Construct validity of the FAQLQ-AF was measured using the Food Allergy Independent Measure (FAIM) [24]. The questionnaire was translated to Hebrew and back translated according to the World Health Organization instructions [25]. Patients were administered the FAQLQ-AF at two time points during the treatment program. The baseline questionnaire was filled out at the start of therapy, during the initial visit. The second questionnaire was completed at the end of the up-dosing period, when patients achieved maintenance, or when treatment was stopped in the cases of failed treatment. Patients who did not answer ≥ 80% of the questions in the FAQLQ-AF were excluded. A control group, consisting of adult patients with food allergy who were not treated with OIT and were followed at the allergy clinic, filled out the FAQLQ-AF at two different time points.

### Statistical analysis

Statistical analyses were performed using SPSS software (version 20; SPSS, Inc, Chicago, Ill). Values were expressed as medians and interquartile ranges (IQRs) unless otherwise indicated, or counts and percentages, as appropriate. Cronbach's α was used to measure the internal consistency and reliability of the FAQLQ-AF and its sub-scales. Validity was assessed by correlating the FAQLQ-AF with the FAIM, using Spearman correlation coefficient. Analysis of nominal variables was done using Chi test. The distribution of numerical variables was examined by Shapiro–Wilk test. Analysis of the changes in QoL scores between different time points and between groups was performed using the Wilcoxon paired analysis and Mann Whitney test, respectively. Estimates with 95% CI are provided. The effect of each continuous and categorical variable on changes in QoL scores was assessed by Mann Whitney test, and multivariate analysis was performed using Linear Regression. A difference of > 0.5 was considered the minimal clinical important difference (MCID) on QoL questionnaires with a 7-point scale [26]. All analyses were 2-tailed and a p-value of less than 0.05 was considered significant.

## Results

### Patient demographics and clinical background

Between May 2014 and January 2022, 64 patients over the age of 18 years began OIT and reached a final disposition of full or partial desensitization or treatment failure. Of these, 44 adults completed the FAQLQ-AF questionnaires before and after treatment and were included in the study. The remaining 20 patients filled the FAQLQ-AF only at a single time point and were therefore excluded. Patients included in the study and those who were excluded were comparable in demographics and clinical characteristics (Table 1s). Excluded patients experienced more epinephrine treated reactions and their OIT failure rate tended to be higher. The 44 OIT-treated patients were compared to 11 adults with food allergy not undergoing OIT. The demographic and clinical characteristics were comparable between the study and control groups (Table 1). Most patients in both groups were males (p = 0.2), and many had multiple food allergy (p = 0.3). The majority had asthma (p = 1.0) and HDM sensitization (p = 1.0) as co-morbidities. The rate of previous anaphylactic reactions to the treated allergen was high (72.7%) and comparable in both groups (p = 0.7). The study group however, had a higher rate of previous use of epinephrine injection, although not statistically significant (p = 0.09). 43.2% of adults were treated for milk allergy, 4.5% for egg, 20.5% for peanut, 13.6% for sesame, 6.8% for walnut, 6.8% for cashew and 4.5% for hazelnut. The median skin prick test was 9 mm and the median starting dose was 23.8 mg of protein (IQR 10–90 mg). The median treatment duration was 10.3 months (IQR 6.3–16.0 months). 65.9% had reactions, requiring epinephrine injection, during up dosing rounds and 22.3% required epinephrine for reactions at home. 75% of patients reached full desensitization. An additional 6.8% reached partial desensitization (median dose 720 mg protein), one due to adverse reactions and two due to pregnancy. 18.2% failed, six due to adverse reactions and two due to emotional difficulties (Table 1).

**Table 1:** Patient demographic and clinical data

Parameter	Oral immunotherapy n = 44	Controls n = 11	p value
Demographics and clinical background			
Gender (Male)	26 (59.1%)	4 (36.4%)	0.2
Age (years)	23.4 (20.4–26.6)	19.8 (19.3- 25)	0.2
Multiple food allergy	15 (34.1%)	6 (54.5%)	0.3
Asthma	26 (59.1%)	7 (63.6%)	1.0
HDM sensitization	37 (84.1%)	9 (81.8%)	1.0
Prior anaphylaxis	32 (72.7%)	7 (63.6%)	0.7
Prior use of epinephrine	26 (59.1%)	3 (27.3%)	0.09
Oral immunotherapy			
Allergen treated			
Milk	19 (43.2%)		
Egg	2 (4.5%)		
Peanut	9 (20.5%)		
Sesame	6 (13.6%)		
Walnut	3 (6.8%)		
Cashew	3 (6.8%)		
Hazelnut	2 (4.5%)		
Skin prick test (mm)	9.0 (7.0–12.5)		
Starting dose (mg of protein)	23.8 (10–90)		
Treatment duration (months)	10.3 (6.3–16.0)		
Epinephrine			
In-clinic	15 (34.1%)		
Home treatment	10 (22.3%)		
Status			
Full desensitization	33 (75%)		
Partial desensitization	3 (6.8%)		
Failure	8 (18.2%)		

Numeric variables are presented as number and percentage

Continuous variables are presented as median and interquartile range

### The change in quality of life following oral immunotherapy

All 55 baseline FAQLQ-AF questionnaires (from 44 OIT-treated and 11 control patients) were used for validation of the Hebrew translation. The internal consistency was high for all domains (AADR = 0.941, EI = 0.891, RAE = 0.853 and FAH = 0.701) and the total score (TS = 0.951). Additionally, the Hebrew translated questionnaires showed medium-strong correlation between the FAIM and each domain (AADR = 0.569, EI = 0.521, RAE = 0.579 and FAH = 0.394, p < 0.001) and for the Total score (TS = 0.590, p < 0.001) (Table 2).

**Table 2:** Correlation and internal consistency between FAQLQ-AF and FAIM, n = 55

FAQLQ-AF	Cronbach α^a^	FAIM	
		Rho^b^	p value
Total score	0.951	0.590	< 0.001
Allergen avoidance and dietary restrictions	0.941	0.569	< 0.001
Emotional impact	0.891	0.521	< 0.001
Risk of accidental exposure	0.853	0.579	< 0.001
Food allergy related health	0.701	0.394	< 0.001

^a^Analysis performed using Cronbach Alpha Analysis

^b^Analysis performed using Spearman correlation coefficient

The study group answered the FAQLQ-AF at the start of OIT and upon reaching maintenance [median, (IQR) 10.3 months (6.2–16.5)]. The control group answered at two different clinic visits [median, (IQR) 8.4 months (4.3–17.2)], and the responses were comparable to one another (p = 0.84). The baseline scores of the study group and the controls were comparable for most domains (AADR, p = 0.48; EI, p = 0.92; RAE, p = 0.49), as well as for the total score, p = 0.54 and FAIM, p = 0.51. The Food Allergy related Health (FAH) domain of the study group was significantly lower (better) at baseline, 3.7 vs 5.3, p = 0.02 (Table 3). After completing OIT, however, the study group demonstrated significantly lower scores in all domains (AADR, p = 0.02; EI, RAE, FAH, total score and FAIM P < 0.01) (Table 3).

**Table 3:** Comparison of FAQLQ-AF scores between oral immunotherapy patients and controls

Item	Baseline				Second time point			
	OIT n = 44	Controls n = 11	Estimate (95% CI)	p value	OIT n = 44	Controls n = 11	Estimate (95% CI)	p value
AADR	5.5 (3.92–5.97)	6.4 (3.50–6.60)	0.4 (− 1.0 to 1.0)	0.44	3.2 (1.50–5.60)	5.7 (3.60–6.30)	1.6 (0.2–3.0)	**0.02**
EI	5.8 (4.70–6.30)	6.0 (4.00–6.60)	0 (− 0.9 to 0.7)	0.92	4.0 (1.92–5.40)	6.1 (3.90–6.90)	1.7 (0.5–3.0)	**P < 0.01**
RAE	5.9 (3.90–5.80)	5.6 (3.10–6.10)	0.2 (− 0.6 to 1.2)	0.49	3.3 (1.40–5.30)	4.9 (3.80–6.50)	1.7 (0.6–3.1)	**P < 0.01**
FAH	3.7 (2.77–5.22)	5.3 (4.70–6.70)	1.4 (0.4 to 2.6)	**0.02**	2.7 (1.70–4.92)	4.7 (3.70–6.70)	2.0 (0.6–3.0)	**P < 0.01**
TS	5.2 (4.02–5.77)	6.2 (3.70–6.40)	0.3 (− 0.7 to 1.1)	0.54	3.25 (1.60–5.10)	5.9 (3.30–6.40)	1.7 (0.4–3.1)	**P < 0.01**
FAIM	4.0 (3.35–4.50)	4.5 (2.20–5.20)	0.3 (− 1.1 to 1)	0.51	2.5 (1.85–4.20)	4.0 (3.00–5.30)	1.3 (0.5–2.3)	**P < 0.01**

Data presented as median and interquartile ranges

OIT: Oral Immunotherapy; AADR: Allergen Avoidance and Dietary Restriction; EI: Emotional Impact; RAE: Risk of Allergen Exposure; FAH: Food Allergy related Health; TS: Total Score; FAIM: Food Allergy Independent Measure

These are the p values that are statisticaly significant

**Fig. 1:**
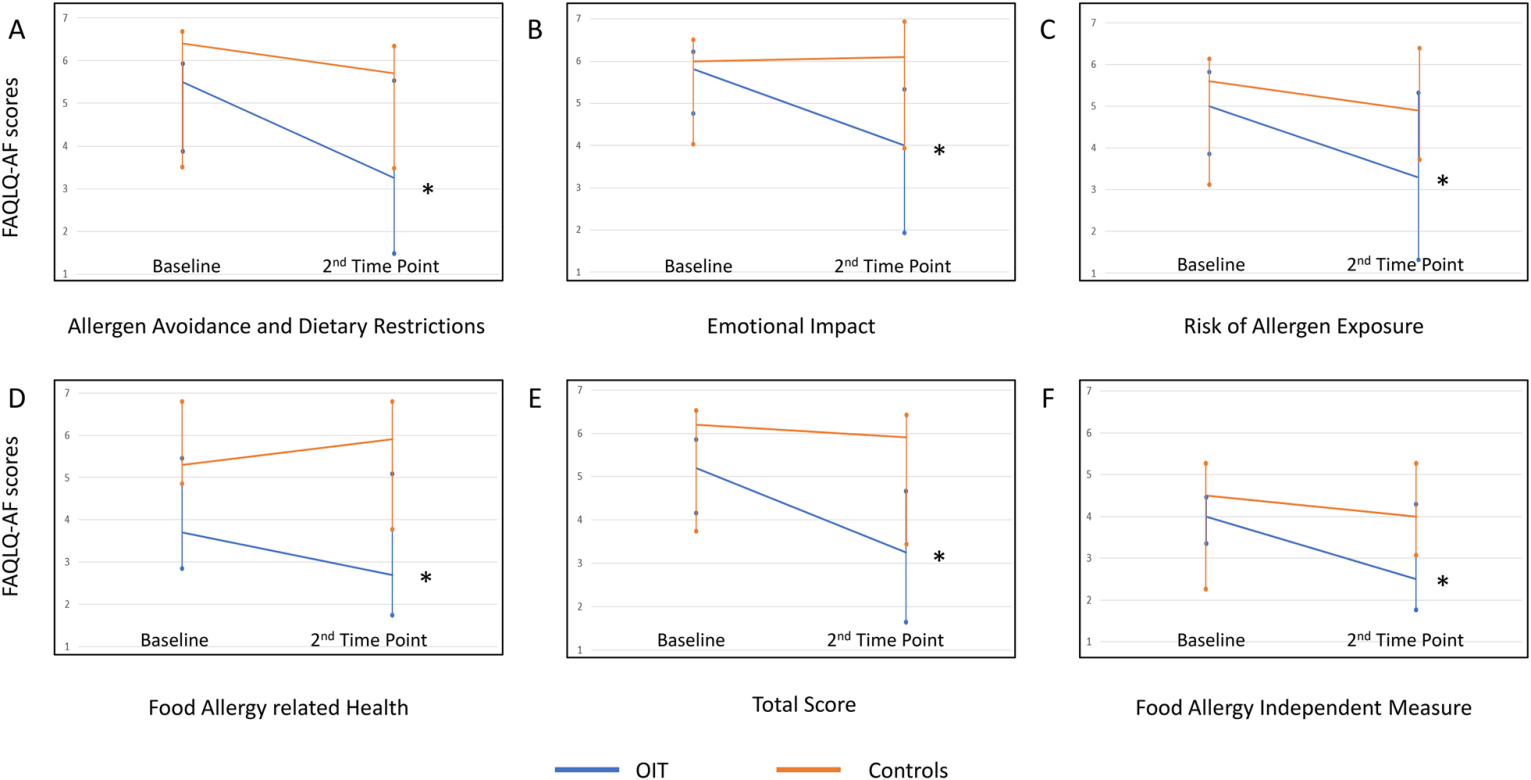
FAQLQ-AF scores in oral immunotherapy in patients undergoing OIT versus controls. Scores are presented as median and IQR. **A** Allergen avoidance and dietary restriction; **B** emotional Impact; **C** Risk of Allergen Exposure; **D** Food Allergy related Health; **E** Total Score; **F** Food Allergy Independent Measure. p values for the OIT group: AADR < 0.001, EI < 0.001, RAE < 0.001, FAH = 0.026, Sum < 0.001, FAIM < 0.001. P values for the controls: AADR = 0.33, EI-0.11, RAE = 0.26, FAH = 0.47, TS = 0.96, FAIM = 0.28

**Fig. 2:**
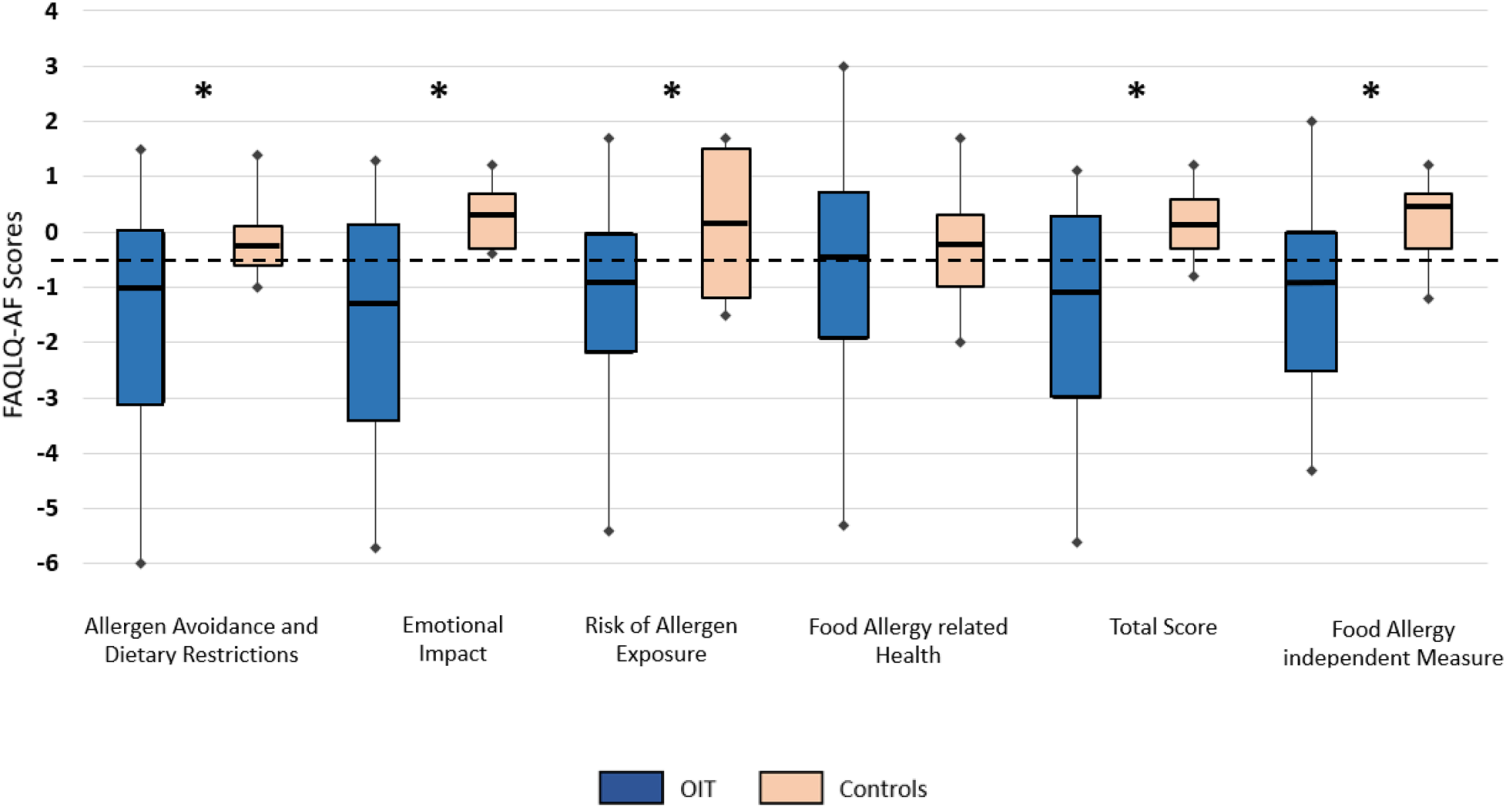
Comparison of the delta in FAQLQ-AF scores between the study group (n = 44) and control group (n = 11). The delta in the study group for all domains but FAH was better than controls both statistically and clinically (represented by the − 0.5 dotted line). AADR: Allergen Avoidance and Dietary Restriction; EI: Emotional Impact; RAE: Risk of Allergen Exposure; FAH: Food Allergy related Health; TS: Total Score; FAIM: Food Allergy Independent Measure. p values for Delta AADR = 0.044, delta EI = 0.002, Delta RAE = 0.012, Delta FAH = 0.54, Delta Total Score = 0.011

The change in FAQLQ-AF scores between start and maintenance was significantly better in the study group in all domains (p < 0.001) (Table 2s, Fig. 1). These changes markedly exceeded the minimal clinically important difference (0.5 points) for all domains as well. The control group did not show a statistically significant change in scores (Table 2s), but a mild clinical improvement (over 0.5 points) was noted in 3 domains: AADR, RAE and FAH, as well as the FAIM. The EI domain in the control group demonstrated worse scores at the second time point, and the total score did not show a clinical improvement (Table 2s). The delta in scores of the study group compared to controls was significantly better in all domains except for FAH (AADR, estimate 0.9, 95% CI 0–2.4, p = 0.04; EI, estimate 1.6, 95% CI 0.6–3.1, p < 0.01; RAE, estimate 1.4, 95% CI 0.3–2.5, p = 0.01; total score, estimate 1.1, 95% CI 0.2–2.4, p = 0.01; and FAIM, estimate 1.4, 95% CI 0.5–2.3, p < 0.01) (Fig. 2).

We next examined the association between clinical and demographic parameters and between a significant change in FAQLQ-AF scores using univariate analysis (Table 3s). Patients without multiple food allergy showed significantly greater improvement in scores in the EI (p = 0.02), the RAE (p = 0.03) and the FAH (p = 0.05) domains, as well as the total score (p = 0.01). Patients who did not use epinephrine during home treatment also showed significantly better improvement in the AADR domain (p = 0.004) and the total score (p = 0.04). Additionally, the status at the end of treatment, was significantly associated with better change in the AADR (p < 0.001), RAE (p < 0.02) domains and the total score (p = 0.004). Being of male gender showed a trend of association with better improvement in scores in the EI (p = 0.08), RAE (p = 0.07), FAH (p = 0.06) domains and TS (p = 0.09) (Table 3s). Linear regression analysis demonstrated that epinephrine use during home treatment showed only a trend with a change in the AADR domain (p = 0.07) but no association with other domains. The lack of multiple food allergy showed significant association to better improvement in scores in the EI (p = 0.007) and RAE (p = 0.05) domains, as well as the total score (p = 0.03) with a trend for improvement in the AADR and FAH domains (p = 0.09 and p = 0.06, respectively). Also, having reached full desensitization had a statistically significant association with changes in the FAQLQ-AF scores, AADR (p = 0.002), and RAE (p = 0.05) domains, as well as the total score (p = 0.01). The association with changes in the EI and FAH domains was statistically borderline (p = 0.06 and p = 0.07, respectively) (Table 4).

**Table 4:** Linear regression analysis for predictors of improved FAQLQ-AF scores from baseline to maintenance

Variable	Correlation				
	Allergen avoidance and dietary restrictions	Emotional impact	Risk of allergen exposure	Food allergy related health	Total score
OIT status					
Coefficient (95% CI)	0.441 (0.4–1.8)	0.262 (− 0.5 to 1.4)	0.295 (0.0–1.5)	0.278 (0.0–1.4)	0.366 (0.2–1.5)
p value	**0.002**	0.06	**0.05**	0.07	**0.01**
Multiple food allergy					
Coefficient (95% CI)	0.215 (− 0.1 to 1.9)	0.376 (0.4–2.6)	0.287 (0.0–2.3)	0.28 (0.0–2.2)	0.298 (0.1–2.1)
p value	0.09	**0.007**	**0.05**	0.06	**0.03**
Epinephrine during home treatment					
Coefficient (95% CI)	0.238 (− 0.1 to 1.9)	0.178 (− 0.4 to 2.6)	0.134 (− 0.7 to 2.0)	− 0.024 (− 1.4 to1.2)	0.185 (− 0.4 to 2.0)
p value	0.07	0.2	0.36	0.87	0.181

These are the p values that are statisticaly significant

## Discussion/Conclusions

This study examined the change in quality of life of adults with food allergy following OIT. The main findings of this study are that adults experience an overall significant improvement in their QoL, especially regarding the emotional and social aspects, but less so when examining food allergy related health. The most important factors impacting improvement were not having multiple food allergy and succeeding in reaching full desensitization.

Data on QoL of adult patients with food allergy is limited. One study, translating and validating the FAQLQ-AF in eight European countries, found a wide variety of QoL scores in adults between the countries. This was attributed to cultural differences in perception of disease severity, practices and traditions of eating and dining out, as well as differences between countries in healthcare, treatment availability and health related costs [27]. Interestingly, our study demonstrates that Israeli adults, both in the study group and even more so in the control group, score worse than in all the eight European countries examined. Our population also demonstrated worse baseline scores than Dutch and American populations [28, 29], showing higher scores for the study group as well as the controls.

While limited data is available on QoL of adults with food allergy, there is no data, to the best of our knowledge, on QoL of adult patients undergoing OIT. OIT has been shown to be successful in desensitizing children [12, 30–32], and to improve both their QoL as well as the QoL of their caretakers(33, 34). These patient-centered positive outcomes contributed to the acceptance of OIT as a formal treatment in FA guidelines worldwide [10, 35]. Recently, we demonstrated that the adult population can succeed in achieving desensitization when treated with OIT [11]. We did find, however, that adults suffer from more severe reactions throughout the process, both in clinic as well as during home treatment. Moreover, the ability to successfully desensitize patients with milk allergy decreases with age, with adults having a significantly higher failure rate. Improving patient QoL is a major goal of OIT and it is therefore extremely important to evaluate the impact of treatment on the QoL of adult patients.

Our main finding is that there is a significant clinical and statistical improvement in patient QoL following OIT in all aspects, emotional as well as dietary restriction and fear of accidental exposure, as depicted by the different questionnaire domains. This was evident both in better scores compared to controls in the second time point, and in a substantial improvement in scores between the two different time points in the study group. Only the FAH domain did not improve. This domain contains three questions dealing with the general perception of health due to FA and fear of having an additional un-diagnosed FA [22]. This domain does not appear in FAQLQ questionnaires in younger ages [36], since it requires a more mature and integrated perception of health. Not surprisingly, in our cohort, adults with multiple FA demonstrated a deterioration in QoL in this domain, while adults with single FA improved. The fact that this was the only domain in which the delta in scores was not significant, proves the complexity of health perception in adults, and the heavy burden carried by multiple FA, even when desensitization was achieved for one of the allergens.

We found that only multiple versus single FA and the ability to reach full or partial desensitization versus treatment failure had a significant impact on the change in QoL during OIT. While the use of epinephrine during home treatment had a significant effect on the emotional impact domain, it was not found to be influential upon multivariate analysis. Other studies examining parameters influencing HRQOL in adults with food allergy showed better scores in adult onset FA versus childhood onset [37], and better scores following negative oral food challenges [38, 39]. However, worse scores correlating with a negative impact on social life [28] and tree-nut and peanut allergy, were noted [40].

It is interesting to note that male patients reported larger improvement in QoL in all domains, including the total score, albeit without statistical significance. Similarly, in a Danish study of FA related QoL differences in scores were demonstrated between genders, showing that females tend to score significantly worse on the FAQLQ questionnaires than males, at all ages (as well as adults) and in all domains [28]. While gender is known to influence the perception of HRQOL [41], it was not shown to be significant in the perception of change in QoL due to OIT and might not be as influential as other parameters with respect to treatment.

This study has several limitations. First, the study group had lower (better) QoL scores at the start of treatment compared to the control group, a finding similar to other studies in younger ages [8, 9] that might reflect the positive influence of a pro-active approach when beginning OIT on patients with FA. We overcame this by analyzing the change in scores in both groups during the study, and showing that only in the study group it was significantly better, both clinically and statistically. Another limitation is the relatively small number of patients, both in the study and control groups. This reflects the low numbers of adults with food allergy [42], particularly those undergoing OIT and emphasizes the lack of data regarding the benefits of treatment in this age group. This study also lacks a long-term follow up evaluation, which would be telling regarding the durability of the described changes in their QOL. Based on a previous report we would expect further improvement [8], and this will be addressed in future studies. Finally, some adults undergoing OIT did not complete the two questionnaires and were therefore excluded. These patients had more in-clinic reactions requiring epinephrine and a higher failure rate. As the improvements in QoL scores in the study population were driven primarily by those who were fully desensitized, including additional patients who failed treatment could have affected the results. However, the main goal of the study was to show that achieving full desensitization, and not merely participating in OIT, improves patients' QoL. This finding is unlikely to be affected by inclusion of excluded patients.

In summary, this study demonstrates that OIT has a beneficial effect on the QoL of adult patients with food allergy. Considering that adults may face a variety of challenges in their personal life during OIT, improvement in QoL shows that the overall benefits of the treatment outweigh the difficulties. These findings, added to previous findings of successful desensitization in this age group, are important in promoting OIT as an effective treatment for the adult population. While in younger patients the beneficial effects of OIT on QoL increases with time [9], taking into consideration the different aspects of chronic health conditions in adults, we believe that future studies need to focus on the long-term effects on QoL in this age group.

## Supplementary Information

Supplementary file 1.

## Data Availability

No datasets were generated or analysed during the current study.
