# Supplementary material for: Oral immunotherapy improves the quality of life of adults with food allergy
Source: Allergy Asthma Clin Immunol. 2024 Oct 14;20:53. doi: 10.1186/s13223-024-00915-6 (PMC11472437; doi:10.1186/s13223-024-00915-6)
Supplement: Supplementary file 1 [file 13223_2024_915_MOESM1_ESM.docx]

**SUPPORTING INFORMATION**

**Table 1s.** Study group versus patients with incomplete FAQLQ-AF- demographic and clinical data

| **Parameter** | | | **Study group**  **n=44** | **Incomplete FAQLQ-AF**  **n=20** | **P value** |
| --- | --- | --- | --- | --- | --- |
| **Demographics and clinical background** | Gender (Male) | | 26 (59.1%) | 12 (60%) | 0.6 |
|  | Age (years) | | 23.4 (20.4-26.6) | 22.5 (19.3- 25.5) | 0.3 |
|  | Multiple food allergy | | 15 (34.1%) | 9 (45%) | 0.3 |
|  | Asthma | | 26 (59.1%) | 14 (70%) | 0.3 |
|  | HDM sensitization | | 37 (84.1%) | 16 (80%) | 0.5 |
|  | Prior anaphylaxis | | 32 (72.7%) | 13 (65%) | 0.4 |
|  | Prior use of epinephrine | | 26 (59.1%) | 11 (55%) | 0.5 |
| **Oral Immunotherapy** | Allergen treated | Milk | 19 (43.2%) | 9 (45%) | 0.7 |
|  |  | Egg | 2 (4.5%) | 1 (5%) |  |
|  |  | Peanut | 9 (20.5%) | 3 (15%) |  |
|  |  | Sesame | 6 (13.6%) | 2 (10%) |  |
|  |  | Walnut | 3 (6.8%) | 4 (20%) |  |
|  |  | Cashew | 3 (6.8%) | 1 (5%) |  |
|  |  | Hazelnut | 2 (4.5%) | 0 (0%) |  |
|  | Skin prick test (mm) | | 9.0 (7.0-12.5) | 8.5 (7- 12.5) | 0.8 |
|  | Starting dose (mg of protein) | | 23.8 (10-90) | 35 (10.5-75) | 0.8 |
|  | Treatment duration (months) | | 10.3 (6.3- 16.0) | 8.35 (4.3- 15.3) | 0.6 |
|  | Epinephrine | In-clinic | 15 (34.1%) | 13 (65%) | **0.02** |
|  |  | Home treatment | 10 (22.3%) | 4 (20%) | 0.5 |
|  | Status | Full desensitization | 33 (75%) | 11 (55%) | 0.2 |
|  |  | Partial desensitization | 3 (6.8%) | 2 (10%) |  |
|  |  | Failure | 8 (18.2%) | 7 (35%) |  |

Numeric variables are presented as number and percentage

Continuous variables are presented as median and interquartile range

**Table 2s**. Change in FAQLQ-AF scores between baseline and second time point

| **Item** | **Oral Immunotherapy (n=44)** | | **P value** | **Controls (n=11)** | | **P value** |
| --- | --- | --- | --- | --- | --- | --- |
|  | **Base line** | **Maintenance** |  | **Base line** | **2^nd^ time point** |  |
| AADR | 5.5 (3.9-5.9) | 3.25 (1.5-5.6) | P<0.001 | 6.4 (3.5- 6.6) | 5.7 (3.6- 6.3) | P=0.32 |
| EI | 5.8 (4.7- 6.3) | 4.0 (1.9- 5.4) | P<0.001 | 6.0 (4.0-6.6) | 6.1 (3.9-6.9) | P=0.11 |
| RAE | 5.0 (3.9- 5.8) | 3.3 (1.4- 5.3) | P<0.001 | 5.6 (3.1- 6.1) | 4.9 (3.8- 6.5) | P=0.26 |
| FAH | 3.7 (2.8- 5.28) | 2.7 (1.7- 4.9) | P=0.02 | 5.3 (4.7- 6.7) | 4.7 (3.7- 6.7) | P=0.47 |
| TS | 5.2 (4.08- 5.8) | 3.25 (1.6- 5.1) | P<0.001 | 6.2 (3.7- 6.4) | 5.9 (3.3- 6.4) | P=0.96 |
| FAIM | 4.0 (3.38- 4.5) | 2.5 (1.8- 4.2) | P<0.001 | 4.5 (2.2- 5.2) | 4.0 (3.0- 5.3) | P=0.28 |

AADR= Allergen Avoidance and Dietary Restrictions; EI= Emotional Impact; RAE= Risk of Allergen Exposure; FAH= Food Allergy related Health; TS= Total Score; FAIM= Food Allergy Independent Measure

| **Variable** | **Group**  **n=44** | **Median (IQR) change in QOL scores from baseline to maintenance** | | | | |
| --- | --- | --- | --- | --- | --- | --- |
|  |  | **AADR** | **EI** | **RAE** | **FAH** | **TS** |
| Gender | Male (n=26) | -1.6  (-4 – - 0.3) | -2.3  (-2.8 – -0.1) | -1.7  (-3.7 – -0.1) | -1.3  (-2.0 – 0.3) | -1.6  (-3.2 – -0.2) |
|  | Female (n=18) | -0.95  (-1.7 – 0.5) | -0.75  (-2.4 – 0.8) | -0.3  (-1.1 – 0.6) | 0.0  (-0.9 – 1.1) | -0.7  (-1.4 – 0.5) |
|  | P value | 0.19 | 0.08 | 0.07 | 0.06 | 0.09 |
| Asthma | No (n=18) | -1.3  (-2.4 – 0.1) | -0.9  (-3.5 – 0.2) | -0.7  (-1.6 – 0.6) | -0.2  (-2.2 – 0.8) | -1.1  (-2.5 – 0.3) |
|  | Yes (n=26) | -0.9  (-3.6 – -0.1) | -1.5  (-3.4 – -0.1) | -1.2  (-3.5 – -0.2) | -0.7  (-1.8 – 0.4) | -1.0  (-3.1 – -0.1) |
|  | P value | 0.98 | 0.66 | 0.35 | 0.92 | 0.58 |
| Multiple Food Allergy | No (n=29) | -1.5  (-4.2 – -0.1) | **-2.3**  **(-3.8 – -0.6)** | **-1.2**  **(-3.9 – -0.2)** | **-0.7**  **(-2.0 – 0.2)** | **-1.4**  **(-3.4 – -0.3)** |
|  | Yes (n=15) | -0.5  (-1.5 – 0.1) | **0.1**  **(-1.8 – 0.6)** | **-0.2**  **(-1.5 – 0.8)** | **0.4**  **(-1.8 – 1.5)** | **-0.3**  **(-1.5 – 0.5)** |
|  | P value | 0.08 | **0.002** | **0.03** | **0.05** | **0.01** |
| Prior Anaphylaxis | No (n=12) | -1.4  (-2.8 – 0.4) | -0.9  (-3.3 – 0.3) | -0.5  (-1.4 – 0.2) | 0.0  (-1.5 – 0.9) | -0.9  (-2.4 – -0.1) |
|  | Yes (n=32) | -0.9  (-3.9 – 0.0) | -1.5  (-3.6 – 0.1) | -1.2  (-3.3 – -0.1) | -0.7  (-2.0 – 0.3) | -1.1  (-3.2 – 0.2) |
|  | P value | 0.71 | 0.56 | 0.29 | 0.17 | 0.44 |
| Epinephrine In- Clinic | No (n=15) | -0.4  (-1.5 – 0.7) | -1.1  (-1.9 – 0.1) | -0.9  (-2.1 – 0.5) | 0.0  (-2.0 – 0.7) | -0.4  (-1.3 – 0.4) |
|  | Yes (n=29) | -1.5  (-3.3 – -0.4) | -2.3  (-3.5 – 0.0) | -1.1  (-2.7 – -0.1) | -0.7  (-1.9 – 0.5) | -1.4  (-3.1 – 0.2) |
|  | P value | 0.11 | 0.52 | 0.83 | 0.90 | 0.38 |
| Epinephrine Home Treatment | No (n=34) | **-1.5**  **(-4.0 – -0.4)** | -2.0  (-3.6 – 0.1) | -1.2  (-3.5 – -0.1) | -0.7  (-2.0 – 0.7) | **-1.4**  **(-3.2 – -0.1)** |
|  | Yes (n=10) | **0.1**  **(-0.6 – 0.6)** | -0.4  (-1.5 – 0.2) | -0.3  (-1.2 – 0.8) | 0.0  (-1.7 – 0.4) | **-0.2**  **(-1.0 – 0.3)** |
|  | P value | **0.004** | 0.09 | 0.17 | 0.61 | **0.04** |
| Status OIT | FD  (n=33) | **-1.7**  **(-4 – -0.7)** | -2.3  (-3.7 – 0.0) | **-1.2**  **(-3.6 – -0.2)** | -0.7  (-1.9 – 0.3) | **-1.4**  **(-3.2- -0.3)** |
|  | PD (n=3) | **-0.4**  **(-1.2 – 0.7)** | -1.9  (-2.9 – 0.4) | **0.0**  **(-0.4 – 1.3)** | 0.0  (-2.0 – 3.0) | **-0.9**  **(-1.1- 1.1)** |
|  | Failure  (n=8) | **0.6**  **(0.2-1.1)** | -0.1  (-0.8-0.5) | **0.1**  **(-1.1-0.8)** | 0.5  (-1.5-1.9) | **0.3**  **(-0.3- 0.8)** |
|  | P value | **<0.001** | 0.063 | **0.02** | 0.17 | **0.004** |

**Table 3s**. The effect of variables before and during OIT on FAQLQ-AF change from OIT baseline to maintenance

AADR= Allergen Avoidance and Dietary Restrictions; EI= Emotional Impact; RAE= Risk of Allergen Exposure; FAH= Food Allergy related Health; TS= Total Score; FAIM= Food Allergy Independent Measure
